# Outcomes and risk factors for failed trial of labor after cesarean delivery (TOLAC) in women with one previous cesarean section: a Chinese population-based study

**DOI:** 10.1186/s12884-022-05005-2

**Published:** 2022-09-03

**Authors:** Xiaobo He, Qiaona Dai, Xiaoli Wu, Junjun Zhou, Jie Li

**Affiliations:** Department of Obstetrics, Ningbo Women and Children’s Hospital, Ningbo, 315012 Zhejiang China

**Keywords:** Cesarean delivery, TOLAC, Risk factor, VBAC, Previous cesarean delivery

## Abstract

**Objective:**

To evaluate the outcomes and risk factors for trial of labor after cesarean delivery (TOLAC) failure in patients in China.

**Methods:**

Consecutive patients who had a previous cesarean delivery (CD) and attempted TOLAC were included from 2014 to 2020. Patients who successfully delivered were classified into the TOLAC success group. Patients who attempted TOLAC but had a repeat CD due to medical issues were classified into the TOLAC failure group. Multiple logistic regression analyses were performed to examine the risk factors for TOLAC failure.

**Results:**

In total, 720 women who had a previous CD and attempted TOLAC were identified and included. The success rate of TOLAC was 84.2%(606/720). Seven patients were diagnosed with uterine rupture, none of whom underwent hysterectomy. Multiple logistic regression analysis showed that the induction of labor (OR = 2.843, 95% CI: 1.571–5.145, *P* < 0.001) was positively associated with TOLAC failure, but the thickness of the lower uterine segment (LUS) (OR = 0.215, 95% CI: 0.103–0.448, *P* < 0.001) was negatively associated with TOLAC failure.

**Conclusions:**

This study suggested that TOLAC was effective in decreasing CD rates in the Chinese population. The induction of labor was positively associated with TOLAC failure, but the thickness of the LUS was negatively associated with TOLAC failure. Our findings need to be confirmed in larger samples with patients of different ethnicities.

## Background

With the promotion of the two- and three-child policy, trial of labor after cesarean delivery (TOLAC) has been increasingly requested by women with a prior cesarean delivery (CD) in China [1]. Because of the high rate of CD in some cities in China [2], the accepted practice of TOLAC was approved by the government to lower the overall CD rates [3, 4]. In the USA, these attempts were highly successful, and the rate of vaginal birth after cesarean delivery (VBAC) remained high from 19.9% in 1990 to a peak of 28.3% in 1996 and 10% in 2010 [5]. There was a slight decrease of the rate of VBAC because of widespread concerns about complications in TOLAC, including uterine rupture [6]. However, a systematic review of these complications by the National Institutes of Health (NIH) indicated that TOLAC is a reasonable option for many women and has encouraged medical institutions to facilitate access to TOLAC [7, 8].

Several factors have been identified that either increase or decrease the likelihood of successful TOLAC, including a previous vaginal delivery, a favorable cervix and obesity [9, 10]. A retrospective case–control study focusing on Israeli women aged 40 years and older showed that the presence of gestational diabetes, induction of labor and higher birth weight were associated with failed TOLAC [11]. While a retrospective cohort study observed no significant association between induction of labor and failure TOLAC in 232 women with no prior vaginal delivery [12]. These factors have been focus on many studies from the USA, Israel, and elsewhere, but close attention should be given to differences in study subjects and obstetrical practices among countries [13, 14]. The rates of TOLAC, associated risk factors and outcomes in China remain unclear. In this study, we aimed to evaluate the success rate of TOLAC and the risk factors for TOLAC failure in patients in China.

## Methods

### Patient identification

**Fig. 1:**
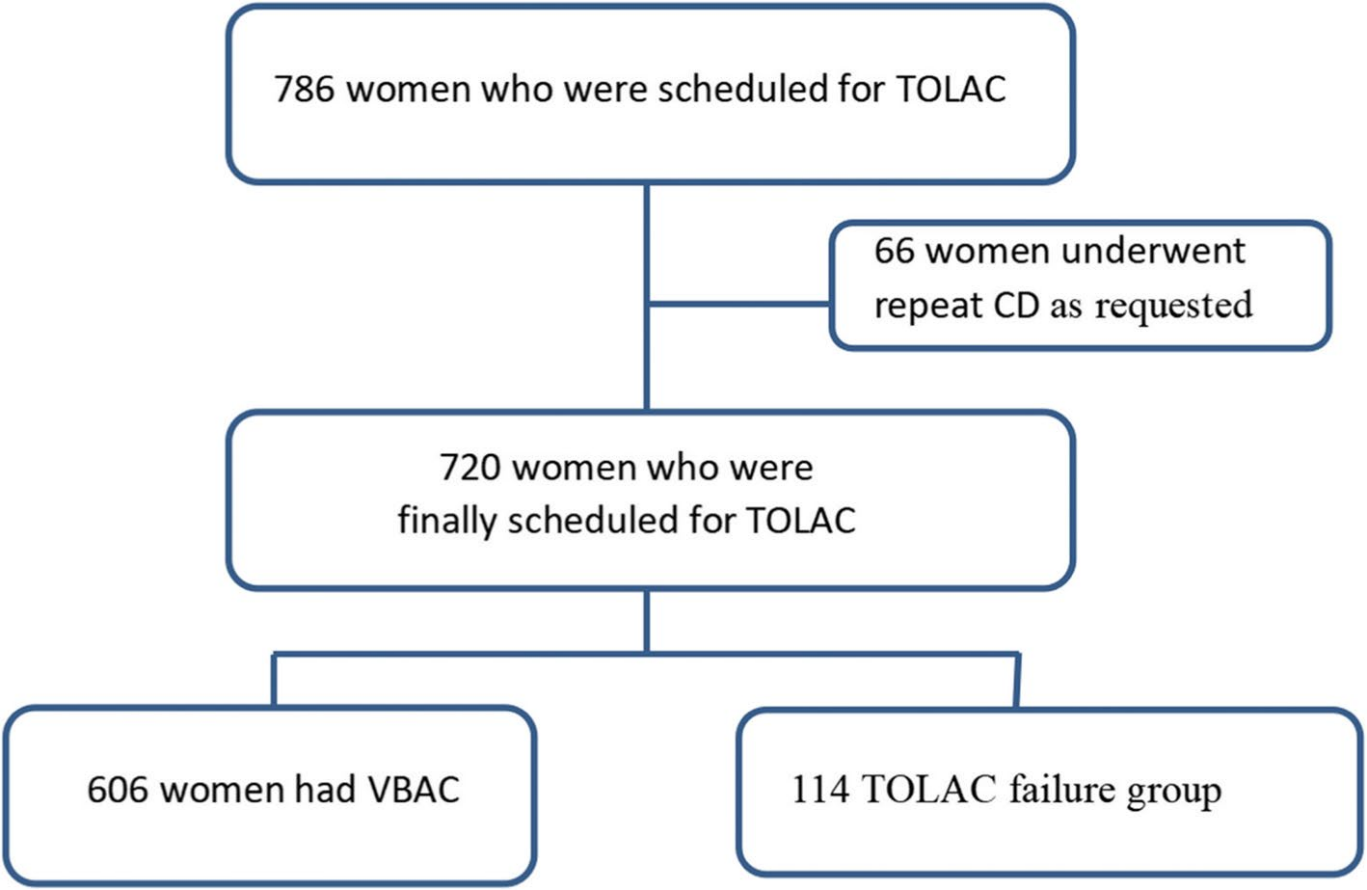
Flow diagram of patient selection. CD = cesarean delivery, TOLAC = trial of labor after cesarean delivery, VBAC = vaginal birth after cesarean delivery

This retrospective study was conducted in accordance with the Declaration of Helsink and approved by the Ethics Committee of Ningbo Women and Children’s Hospital (approval number: EC2017-003).The records/information of all women were anonymized and recorded before analysis. All of the participants provided written informed consent. Information related to maternal history and delivery was extracted from medical and surgical records. All women who met the inclusion criteria between December 2014 and December 2020 were included in the study. The inclusion criteria were as follows: 1) singleton delivery; and 2) had a previous CD but was scheduled for TOLAC. The exclusion criteria were as follow: 1) two or more previous CDs; 2) twin pregnancy; 3) contraindications to vaginal birth (breech presentation, placenta previa); 4) history of other uterine incisions such as myomectomy, “T”, “J” or vertical incisions; 5) incomplete medical records; and patients request for repeat CD during labor (Fig. 1).

### Data collection

The study examined demographic, obstetric, maternal, and delivery variables. These variables were chosen based on factors that were previously linked to TOLAC in other studies and their availability in our current clinical database [9–12, 15, 16]. The demographic variables included age, body mass index in the admission for TOLAC, gravidity, abortion; history of vaginal delivery, gestational weeks at pregnancy termination, estimated birth weight; The clinical factors includes: time from previous CD, indication for previous CD (failed trial of labor) and lower uterine segment (LUS) thickness. The maternal and neonatal outcomes included blood loss during delivery, intrapartum blood transfusion, puerperal infection, NICU admission, Apgar score (5 min, ≤ 7), injury to the bladder and incomplete or complete rupture of the muscle layer of the lower uterus. Before start of TOLAC or CD, the LUS thickness was assessed using an abdominal probe. A normal LUS for ultrasonography is a 2-layer structure consisting of an echogenic layer (the bladder wall) and a usually less echogenic layer (the myometrium). Transabdominal assessments were performed when a woman had a full bladder; the LUS was examined longitudinally and transversely to detect areas of significant uterine scarring and to measure the myometrium. All measurements were made without contractions. Indication for the previous CD (failed trial of labor) was that pregnant women who underwent prior CD because of the failure of labor, not due to other reasons such as maternal request.

If spontaneous labor has not occurred, the induction of labor will be performed according to the recommendation and the medical center’s protocol. The indications for induction of labor included term pregnancy (defined as pregnancy over 40 weeks gestation), gestational diabetes mellitus, gestational hypertensive disorders, oligohydramnios or another medical indication warranting induction of labor. The Bishop score was calculated using the first digital cervical examination at the time of admission. According to the recommendation of induction of labor, women with a cervix Bishop score ≥ 5 or having premature rupture of membrane were directly induced with intravenous oxytocin. Women with a cervix Bishop score < 5 would be performed a transcervical Foley balloon catheter inflated with 70 ml of 0.9% sodium chloride solution to promote cervical ripening. After the balloon was expelled spontaneously or withdrawn after 24 h, artificial rupture of membranes or intravenous oxytocin was initiated.

Blood loss during delivery was defined as the total volume of blood exceeding 1000 ml from the beginning of the cesarean delivery procedure to 24 h later or exceeding 500 ml from the beginning of the vaginal delivery to 24 h later. Puerperal infection was defined as a fever that began on or after postoperative day (POD), and the temperature was more than 37.8℃on 2 successive measurements or greater than 39℃once. Complete rupture of the muscle layer of the lower uterus was defined as a full thickness tear of the uterine wall that also includes uterine serosa (overlying peritoneum). Incomplete rupture of the lower muscle layer of the uterus, known as uterine scar dehiscence, was defined as incomplete uterine scar separation with intact serosa, sometimes referred to “uterine window”. Intrapartum blood transfusion: women were received a transfusion of RBC, plasma or platelets due to acute blood loss, postpartum blood loss and surgery related. Injury to the bladder: urinary bladder injury was defined as any compromise of the bladder wall including full-thickness injury and partial-thickness disruption [17].

### Statistical analysis

The Mann–Whitney U test was used to examine continuous variables, and Fisher’s exact test or chi-squared test was used to examine categorical variables. Continuous variables were presented as means ± SD and categorical variables as median and interquartile range(IQR). All continuous predictors were assumed to be linearly associated with the outcome. Multiple logistic regression analyses were performed to identify the factors for TOLAC failure, in which factors such as gestational weeks at pregnancy termination (> 39^0/7^wks vs.37^0/7^ ~ 38^6/7^wks), and induction of labor (yes vs. no) were entered as categorical variable, and factors such as LUS thickness as continuous variables. All statistical analyses were performed using SAS version 9.3 (SAS Institute, Inc., Cary, NC). Statistical significance was defined as *P* < 0.05.

## Results

During the study, 786 women who attempted TOLAC were identified. Sixty-six participants were excluded because they received repeat CD at their own or their family’s request rather than medical indication during labor. Of the remaining 720 women participants, 606 women underwent TOLAC and had successful VBAC with their next pregnancy, while 114 women underwent TOLAC and had failure VBAC. The rate of successful VBAC among these women was 84.2% (Fig. 1). The characteristics of all patients are shown in Table 1. No significant differences in age, body mass index, gravidity, abortion, history of vaginal delivery, time from previous CD and indication for previous CD (failed trial of labor) were observed between the TOLAC success and TOLAC failure groups. The rate of induction of labor was significantly higher in the TOLAC failure group than in the TOLAC success group (65.8% vs. 29.0%, *P* < 0.001). The gestational weeks at pregnancy termination and estimated birth weight were significantly higher in the TOLAC failure group than in the TOLAC success group (*P* < 0.001, *P* = 0.046). The LUS was significantly thinner in the TOLAC failure group than in the TOLAC success group (*P* < 0.001).

**Table 1:** Demographic and clinical characteristics of the patients in the TOLAC success and failure groups

Clinical parameters	Success group (*n* = 606)	Failure group (*n* = 114)	*P value*
Maternal age (years),	31.21(17–40)	31.86(18–41)	0.769
BMI (kg/m2)	25.40 ± 3.51	25.63 ± 4.61	0.745
Gravidity, (times),	2 (0–9)	3 (0–9)	0.065
Parity, (times),	1 (0–3)	1 (0–3)	0.056
Number of abortions, (times),	1 (0–9)	2 (0–5)	0.643
History of vaginal delivery (no), n (%)	489 (80.69%)	105 (92.10%)	0.093
Gestational weeks at pregnancy termination, (weeks)	39^0/7^(37^0/7^–40^0/7^)	39^5/7^(38^6/7^–40^1/7^)	< 0.001
37^0/7^–38^6/7^	127(20.96%)	48(42.11%)	
≥ 39^0/7^	479(78.22%)	66(57.89%)	
Induction of labor (yes), n (%)	176 (29.04%)	75 (65.79%)	< 0.001
Estimated birth weight (kg)	3.28 (2.84–3.50)	3.35 (3.14–3.66)	0.046
Time from previous CD (years)	6.01 (4.12–8.13)	6.33 (4.39–7.23)	0.642
Indication for the previous CD (failed trial of labor), n (%)	101 (16.80%)	24 (21.12%)	0.534
LUS thickness, mm	22 ± 11	17 ± 10	< 0.001

Data in the table are presented as n (%), mean ± SD, and median [interquartile range]. *CD* Cesarean delivery, *BMI* Body mass index, *LUS* Lower uterine segment

The clinical outcomes of the TOLAC failure and success groups are presented in Table 2. There were 7 patients who had incomplete or complete rupture of the muscle layer of the lower uterus in the success group (rupture of the uterus). There were no significant differences in puerperal infection, bladder injury, or 5-min Apgar scores between the two groups. The volume of blood loss during delivery and the incidence of blood transfusion during delivery in the TOLAC success group were significantly lower than those in the TOLAC failure group. The rate of NICU (neonatal intensive care unit) admission was significantly higher in the success group than in the TOLAC failure group (*P* < 0.001).

**Table 2:** Clinical outcomes of the patients

Clinical outcomes	Success group (*n* = 606)	Failure group (*n* = 114)	*P*
Blood loss during delivery (ml)	300 ± 52	500 ± 60	< 0.001
Intrapartum blood transfusion, n (%)	0 (0)	3 (2.63%)	< 0.001
Puerperal infection, n (%)	3 (0.50%)	0	0.452
Apgar score(5 min, ≤ 7), n (%)	12 (2.00%)	0	0.130
NICU admission, n (%)	96 (15.84%)	6 (5.31%)	< 0.001
Incomplete or complete rupture of the muscle layer of the lower uterus, n (%)	7(0.99%)	0	0.249
Injury to the bladder, n (%)	0	0	

Data in the table are presented as n (%), mean ± SD, and median [interquartile range]. *NICU* Neonatal intensive care unit

Multiple logistic regression analysis showed that the induction of labor (OR = 2.843, 95% CI: 1.571–5.145, *P* < 0.001) was positively associated with TOLAC failure. In contrast, the thickness of the LUS (OR = 0.215, 95% CI: 0.103–0.448, *p* < 0.001) was negatively related to TOLAC failure. The gestational weeks at pregnancy termination (> 39^0/7^wks *vs. 3*7^0/7^ ~ 38^6/7^wks) showed no significant relationship with TOLAC failure (odds ratio [OR] = 3.046, 95% confidence interval [CI]: 0.962–9.642, *P* = 0.055) in Table 3.

**Table 3:** Multiple regression logistic to identify the predictive factors for TOLAC failure in women

	OR	95% CI	*P*
Gestational weeks at pregnancy termination(> 39^0/7^wks vs.37^0/7^ ~ 38^6/7^wks)	3.046	0.962–9.642	0.055
Induction of labor (yes vs. no)	2.843	1.571–5.145	< 0.001
LUS thickness	0.215	0.103–0.448	< 0.001

*OR* Odds ratio, *CI* Confidence interval

## Discussion

The main finding of this study is that the success rate of TOLAC was 84.2% in this study. Patients in the TOLAC success group had a lower volume of blood loss, lower blood transfusion rate, but higher NICU admission rate, compared to the TOLAC failure group, which had the similar results in Caroline’ study [18]. Generally, TOLAC is a potential strategy to reduce CD rates, and successful trials of labor can reduce the incidence of some important adverse outcomes. We observed that TOLAC was safe and feasible for patients who had a prior CD in our hospital.

Uterine rupture is a serious complication of TOLAC. The incidence of this complication among women who attempted TOLAC in the present study was 0.97% (7/720). Other studies reported that the rate of uterine rupture was 1.0 to 4.2% [19–21]. In our study, five patients were diagnosed with hematoma around the uterine scar by ultrasound, and they all recovered after conservative treatment. Two of them complained a slight abnormal abdominal pain after delivery and presented a 6–7 cm hematoma around the uterine scar by ultrasound immediately. Three of them without abnormal complaint found a 2–3 cm hematoma around the uterine scar by ultrasound in the first day of delivery. Two patients had been found omentum majus in the vagina and underwent emergency laparotomy and uterine repair. None of these patients underwent a hysterectomy. In a previous study, the rate of hysterectomy in patients who were scheduled for TOLAC was 3.2/1 000 [22].

In the present study, the univariate analysis revealed that the rate of induction of labor was significantly lower in the TOLAC success group than in the TOLAC failure group. Multiple logistic regression analysis showed that induction of labor was positively associated with TOLAC failure. Cohort studies conducted in India and Israel also showed that labor induction was independently associated with failed TOLAC [9, 15, 23]. The rate of IOL was also higher (16.7%) in the VBAC group in Caroline’ study [18]. When labor begins spontaneously, decisions are easier to be made in TOLAC than when IOL is indicated because of the risk of uterine rupture. In Gabriel’study [16], the IOL also have a higher failed TOLAC delivery rate in grand‑multiparous women. A retrospective case–control study of all women who was aged 40 years and older with a history of previous cesarean delivery showed that induction of labor was more common in the failed TOLAC group [11]. These could be found that association is causally related to failed TOLAC. However, in order to draw reliable conclusions, more prospective cohort studies with large sample size or high-quality randomized clinical trials should be conducted.

In this study, the thickness of the LUS (OR = 0.215, 95% CI: 0.103–0.448, *P* < 0.001) was negatively related to TOLAC failure, meaning that if the LUS is thinner, the opportunity for successful TOLAC is lower, which was similar to the results of Bujold’s study [24]. However, some studies have observed different results, noting that the thickness of the uterine scar was not related to the success of TOLAC [25–27]. A possible reason for this difference is that the accuracy of B-ultrasound in evaluating the thickness and continuity of the uterine scar myometrium is still controversial [28]. According to our study, however, pregnant women with a thinner LUS may be not suitable for TOLAC and can choose elective repeat cesarean delivery (ERCD).

Our results showed that gestational age at delivery was significantly greater in failed TOLAC, there is no significant in multivariate analysis. Similar results were reported in Gabriel’ study that gestational age at TOLAC was lower in the success group but no significant in multivariate analysis [16]. A greater gestational age as a risk factor for failed TOLAC was needed to confirm. Besides, this study did not find any significant difference in the rate of successful TOLAC between groups with regard to indications for previous CD (failed trial of labor), time from previous CD or history of vaginal delivery. However, some literatures found the opposite results [29, 30]. Gabriel et al. found that TOLAC had a very-high success rate among grand-multiparous women [16]. Then, more studies are needed to address the implications of these factors.

There are several limitations to this study. First, with regard to patients with TOLAC failure, it is noteworthy that no uterine rupture was found. The results of uterine dehiscence recorded on the surgical record might be lacking, given that this was a retrospective study with a small number of cases from a single center. Second, many women who would be good candidates for TOLAC still choose ERCD, than would be made the success of TOLAC lower. Further randomized, controlled trials and multicenter studies are required in the future. Third, all patients in this study were Chinese. Further studies in other populations are required to confirm our findings. Finally, data on several confounding factors, such as tobacco use, alcohol consumption, nutritional status, and socioeconomic conditions, were not available in this study, which might lead to deviation from the real TOLAC rate, including overestimation and underestimation. Therefore, our results should be interpreted with caution.

## Conclusions

Our study adds to the limited evidence that TOLAC is effective in decreasing CD rates in the Chinese population. The induction of labor was positively associated with TOLAC failure, but the thickness of the LUS was negatively associated with TOLAC failure. Our findings need to be confirmed in larger samples with patients of different ethnicities.

## Data Availability

The datasets generated and/or analyzed during the current study are available in Clinical trial registration: researchregistry7416 (https://www.researchregistry.com/register-now#home/registrationdetails/61a68ec6e1410e0020396cdb/).
